# Impact of Technical-Tactical and Physical Performance on the Match Outcome in Professional Soccer: A Case Study

**DOI:** 10.5114/jhk/185933

**Published:** 2024-07-17

**Authors:** Benjamin Barthelemy, Guillaume Ravé, Karuppasamy Govindasamy, Ajmol Ali, Juan Del Coso, Julien Demeaux, Benoit Bideau, Hassanel Zouha

**Affiliations:** 1 Movement, Sport, Health and Sciences laboratory (M2S), UFR-STAPS, University of Rennes 2-ENS Cachan, Rennes Cedex, France.; 2 Toulouse Football Club, Toulouse, France.; 3 Department of Physical Education and Sports Sciences, College of Science and Humanities, SRM Institute of Science and Technology, Kattankulathur, Tamilnadu, India.; 4 School of Sport, Exercise and Nutrition, Massey University, Auckland, New Zealand.; 5 Sport Sciences Research Centre, Rey Juan Carlos University, Fuenlabrada, Spain.; 6 Institut International des Sciences du Sport (2IS), 35850, Irodouer, France.

**Keywords:** sports science, match analysis, performance, team sport, elite athlete

## Abstract

Match outcomes and championship rankings are the consequence of the team’s technical, tactical, and physical variables. This study aimed to compare physical and modern technical-tactical performance variables between matches with different outcomes for a professional soccer team. Total distance covered, distance covered between 20.0 and 25.0 km/h and at > 25.0 km/h, distance covered at ≥ 3 m/s^2^ and at ≤ −3 m·s^−2^ along with several modern technical-tactical variables (expected goals in favor (xG) and against (xGA), expected goals chain in favor (xGC) and against (xGCA) and passes per defensive action (PPDA)) were collected for 71 soccer matches during the 2020/2021 and 2021/2022 seasons from a team competing in the French Ligue 2. These technical-tactical and running performance variables were obtained by a validated video tracking system (OPTA) and their values per match were compared depending on the match outcome which was categorized as “loss” (L), “draw” (D) or “win” (W). No significant differences were observed for the different running metrics depending on the match outcome. However, significant differences were observed for xGA (0.70 ± 0.39 vs. 1.24 ± 0.59; p_bonferroni_= 0.004) and xGCA (5.38 ± 2.78 vs. 10.92 ± 6.18; p_bonferroni_ = 0.002) between wins and losses, respectively. Additionally, there was a weak, but significant correlation between xGCA and distance covered in acceleration (r = 0.255; p = 0.032) and deceleration (r = 0.237; p = 0.047). In conclusion, while our study found associations between technical-tactical variables and match outcomes, causality cannot be inferred. Improved technical-tactical performance may positively impact the match result, especially by the reduction of the opposing team's goal expectancy. On the contrary, running performance variables showed no associations with the match outcome.

## Introduction

The most popular sport in the world, a soccer game between two teams, ends with one of the three possible outcomes: draw (D), win (W) or loss (L). The match outcome and more globally, the championship ranking, is the consequence of team performance (Longo et al., 2019). Team performance is the product of the combination of technical, tactical, and physical abilities during a soccer game (Stølen et al., 2005), although a myriad of intrinsic and extrinsic factors can also influence team’s success in a soccer match. From a holistic approach, soccer performance is a complex construct characterized by high dynamicity and complexity as players’ and team’s behaviors must adapt continuously to the continuous performer-environment interactions. Additionally, complexity in this team sport is higher as players have different roles depending on the pitch zone and when they attack or defend. Scoring a goal is the most obvious representation of soccer success and for this reason, the study of the teams’ actions that lead to a goal has been the topic of numerous investigations (Mićović et al., 2023). However, most actions in soccer do not end in a goal, and thus, the final match outcome does not always represent an objective categorization of teams’ performance during a match. In this regard, the study of accumulated statistics and running metrics in matches with different outcomes may be a more robust method to assess the overall team’s performance, instead of investigating only goal-scoring actions. In fact, the rapid evolution of match analysis methods in the past decade has significantly enhanced our understanding of soccer performance. These advancements have allowed for the identification of key performance factors that are closely linked to the likelihood of victory (Lepschy et al., 2021). Additionally, the collection of a high quantity of players’ data during soccer matches helps the creation of complex match statistics to assess the collective behavior of the team in real time to better understand soccer at the professional level (Link et al., 2016). Although linking of several physical, technical and tactical variables with soccer performance, by the comparison of successful and unsuccessful teams (Brito de Souza et al., 2019) or within the same team with different match outcomes (Aquino et al., 2020), has been the topic of numerous previous investigations, the different methodological approaches used and variables assessed impede the unequivocal understanding of which variables are most important to obtain victory.

Today, physical performance during professional soccer matches is routinely assessed with video tracking systems (Buchheit et al., 2014; Castellano et al., 2014; Radziminski et al., 2022) to obtain metrics such as total distance covered, acceleration/deceleration counts or distances, and distance covered at various running velocities, among other variables (Datson et al., 2017). It is worth noting that, since FIFA's approval, GPS tracking has also become highly prevalent, expanding the methods used to measure performance (Abbott et al., 2018). However, the relationship between running performance metrics and victory in professional soccer is still controversial as the studies that have compared running demands of the same team when winning, drawing, and losing a match have found different outcomes. For example, different authors showed higher running performance in wins (Aquino et al., 2017, 2020; Gonçalves et al., 2021). In the German Bundesliga attacking players from winning teams covered a greater distance above 21 km/h compared to draws and losses (Chmura et al., 2018) and the distance covered in kilometers was considered the most influential variable for winning a match (Schauberger et al., 2018). In contrast, other research showed higher running performance during losses, especially for high-intensity running and acceleration, compared to running data when drawing and winning (Augusto et al., 2022). Another study found that both successful and unsuccessful teams presented similar running performance (Asian-Clemente et al., 2019). Overall, these data indicate that higher running performance during a match is not likely a decisive factor for obtaining a victory. Interestingly, a study by Hoppe et al. (2015) showed that it was the distance covered with the ball that was directly related with soccer performance as it reflected the actions performed with the possession of the ball. Collectively, all this information indicates that more investigation is needed to understand the relationship between running performance and success in professional soccer, especially in actions performed with ball possession.

The percentage of ball possession, the number of successful passes, shots at the target, crosses, and successful dribbles describe generally the technical-tactical performance indicators of a soccer team (Asian-Clemente et al., 2022; Aquino et al., 2021; Arjol-Serrano et al., 2021; Bradley et al., 2011; Paul et al., 2015; Sarmento et al., 2014). Among them, shooting quantity and accuracy as well as passing accuracy are the technical-tactical indicators more associated with better teams’ performance (Brito Souza et al., 2019). Recently, new and more complex indicators of technical-tactical performance in soccer have appeared including expected Goals (xG), expected Goals Chain (xGC) and Passes Per Defensive Actions (PPDA) (Eggels et al., 2016; Fernández de la Rosa, 2022; Lucey et al., 2015; Trainor, 2014). These indicators of technical-tactical performance are increasingly popular and used by the technical staff of professional teams to assess the capacity of their own team and the rival to create shooting actions with high probabilities of scoring (Mead et al., 2023). Specifically, the metrics associated with the expected goals created by their own team have been considered a better way of dealing with the randomness in soccer than, for example, a traditional goal-based metric since a shot is a much more common event than a goal (Mead et al., 2023). In this context, all expected goal models predict the likelihood that a given shot will result in a goal through distance to the goal angle and the shot type (although there are much more complex models, but these metrics are often unable to predict performance of the best teams (Rathke, 2017)). While the topic about how expected goal metrics might influence the likelihood of a shot ending in a goal has been well studied (Kharrat et al., 2020), to our knowledge, there is no peer-reviewed research examining the relationship between these technical-tactical indicators and the match outcome in professional soccer.

Therefore, this study aimed to compare physical and new technical-tactical performance variables between matches with different outcomes over two seasons for a soccer team playing in the French Ligue 2 championship during the 2020/2021 and 2021/2022 seasons. The research aimed to establish a relationship between key performance measures and the match outcome to enhance our understanding of the interplay between physical and technical-tactical aspects of the game and success. This study addresses gaps in existing research by investigating how these factors interact and influence match outcomes, particularly by including modern variables that have received limited researchers’ attention.

We formulated the following hypotheses based on our research objectives and relevant literature (Augusto et al., 2022; Mead et al., 2023): i) there would be no significant differences in physical performance variables between matches with different outcomes; ii) there would be significant differences in technical-tactical performance variables between matches with different outcomes.

## Methods

### Participants and Match Sample

The current investigation represents a descriptive, comparative analysis of technical-tactical and running performance metrics of one professional soccer team when it won/drew/lost official matches played over two consecutive seasons. The analyses of technical-tactical performance and physical performance variables were carried out in 71 matches of the same team playing in the French Ligue 2 championship during the 2020/2021 and 2021/2022 seasons. An *a priori* sample size estimation for ANOVA of repeated measures, within factors, with a power of 0.90 (90%), an alpha error of 0.05, and an estimated effect size of 0.5 was performed using G*Power software (Faul et al., 2009). This calculation reported that a minimum of 16 matches had to be analyzed, but we obtained data from all the matches performed over two consecutive seasons (i.e., 71 in total) to ensure that our sample size was sufficiently representative to detect significant differences in the analyzed performances. The team was ranked in the top three of the French Ligue 2 championship in both seasons and the analysis represents behaviors of a successful team. During the 71 matches analyzed, we used the data of the outfield players who played the entire match (n = 605) and they were accumulated to obtain a team’s data per match. This deliberate selection criterion allowed us to avoid differences in freshness between the starters and the players who come on during the match, which could have influenced players’ performance, as substitution moments are specific to each coach's strategy and may be decided for various reasons (Bradley and Noakes, 2013). The study was conducted under the approval of the Medical Ethics Committee of the Toulouse Football Club, Toulouse, France (approval code: 03-2021; approval date: 17 September 2021) and all procedures were undertaken according to the Declaration of Helsinki. During each match, the technical-tactical and physical performances, along with the outcome were measured and analyzed as follows.

### Technical-Tactical Performances Measure

The technical-tactical data (xG, xGC, PPDA) for each match were provided by a video tracking system (OPTA client system, SportVU 2.0). The software used by OPTA generates live match statistics from every action that is taken on the ball, through a combination of human annotation, computer vision and artificial intelligence modelling. The data collection method employed by OPTA (OPTA client system, SportVU 2.0) demonstrates a strong degree of inter-operator reliability, as indicated by Kappa values (>0.86), ICC (>0.88), and low standardized typical errors (varied from 0.00 to 0.37) that meet acceptable standards. Consequently, the data can be considered valid for academic research purposes (Ju, 2022; Liu et al., 2013).

#### Expected Goals (xG)

Expected Goals (xG) is the most popular metric to estimate the probability of a shot ending in a goal based on different factors describing the shot (Fernández de la Rosa, 2022). The xG calculations used spatial and temporal information presented in Table 1 (Anzer and Bauer, 2021). xG was more appropriate than traditional data (shots, shots at the target) to predict the future goal ratio for the rest of the season after a certain number of games played (Anzer and Bauer, 2021).

**Table 1:** Factors used to determine xG value.

Factors	Description
Shot location	The x, y and the z-coordinates of the ball at the time of the shot are used for several features, such as the angle and the distance to the goal center
Speed of the player taking the shot	The running speed of the player attempting the shot, at the time of the shot (in km/h)
Defenders in the line of the shot	The number of defenders in the line of the shot.
Goalkeeper position	The position of the goalkeeper is used for two different features: describing whether they are in the line of the shot and their distance to the goal.
Pressure on the player taking the shot	Various metrics describing the pressure that the player was under while attempting the shot, at the time of the shot
Type of the shot	Describing the body part used for the shot (a head, a leg or other)
Taker ball-control	Describes how the player taking the shot gained control of the ball before/when taking the shot (volley, control shot, dribbling <10m, dribbling >10m, set piece)
After freekick	Indicates whether the shot followed a freekick
Freekick	Describes whether the shot is a direct freekick or not

xG value given to the shot (between 0 and 1) is dependent on the factors presented above and corresponds to the average of the previous shots made in the same situation labeled by the information whether the shot ended up in a goal (1) or not (0).

In contrast to xG, which defines the probability that an offensive shot will end in a goal, the expected goal against (xGA) metric defines the same thing when the opposing team attempts a shot, namely the probability of conceding a goal by the defending team, following an opposing shot. For both variables, the data used for analysis were the accumulated statistics generated by the team in the whole match, assuming that a higher xG or xGA referred to teams with a higher combination in the number of shots and in the likelihood of scoring of these shots.

#### Expected Goal Chain (xGC)

To complement xG, another metric has appeared in modern soccer analysis, the xGC. This metric is defined by the number of xG in which a player has participated. If a player participates in an action that ends in a shot, the xG of the action will be added to this metric for this player (Shank, 2017). This variable gives values to players who are part of passing sequences that end in a shot, and not only consider the player who performs the shot (as is the case with xG). In this context, a higher xCG would be obtained for a combination of passes that ended in a shot, and shots with a higher likelihood of ending in a goal. We denote by xGC the offensive expected goal chains and xGCA the expected goal chains during an opposing shot.

#### Passes Per Defensive Actions (PPDA)

The PPDA metric is calculated by dividing the number of passes performed by the attacking team by the total number of defensive actions performed, i.e., tackles, interceptions, and fouls (PPDA = number of passes performed by the attacking team / number of defensive actions by the defending team) (Trainor, 2014). Both values are calculated with reference to a specific area of the pitch, e.g., in the opponent’s final 60% of the pitch (Trainor, 2014). This metric provides an objective measurement of defensive efficacy as it is calculated with reference to the number of times it is possible for the defensive team to intercept the ball on all opposing passes in the specified area of the pitch where the opposing team may produce an offensive action (Trainor, 2014).

### Physical Performances Measures

The data of running performance metrics were also recorded by the SportVU 2.0 (Statsperform) video tracking system. SportVU 2.0 incorporates an optical tracking system technology that delivers performance statistics by extracting the spatial-time coordinates of players and the ball with statistical algorithms. The total distance (TD) covered (m), the running distance covered (m) between 20.0 and 25.0 km/h (TD 20–25), the running distance covered (m) at a velocity greater than 25.0 km/h (TD>25), the running distance covered (m) at a velocity greater than 20 km/h (TD>20), the running distance covered (m) in acceleration (D-ACC) and the running distance covered (m) in deceleration (D-DEC) were the physical variables measured. Both acceleration and deceleration variables considered the movements made with an intensity at ≥ (+) or ≤ (−) 3 m/s^2^, as previously suggested (Miguel et al., 2021).

### Match Outcome

The match outcome was analyzed according to the match status at the end of the match and categorized as “loss” (L), “draw” (D) or “win” (W).

### Statistical Analysis

For all analyses, Jamovi software 2.0.0.0 was used, and the level of significance was set at *p* = 0.05. First, the variables were checked to verify their conformity with a normal distribution (Shapiro-Wilk; w ranging from 0.966 to 0.982 for all variables), and a descriptive analysis was carried out by calculating the mean, standard deviation, for the parametric data over all the matches, and median and range for non-parametric data. One-way analysis of variance (ANOVA) was used to compare values of technical-tactical and running performance variables in the different match outcomes. *Post-hoc* analyses were performed using the Bonferroni test to detect differences in technical-tactical and physical variables for the pairwise comparison of the team when it won vs. drew vs. lost the match. Pearson's correlation (r) and 95% confidence interval were calculated and used to assess the relationships between technical-tactical data and physical performance data. A correlation coefficient between 0 and 0.3 was considered weak, between 0.4 and 0.6 was considered moderate, and greater than 0.7 was considered strong (Akoglu, 2018). This calculation was performed using all the pool of data, irrespective of the match outcome.

## Results

Of the 71 games analyzed, there were 40 wins, 18 draws and 13 losses. The physical performances and the technical-tactical performances of all 71 matches according to the outcome are presented in Table 2. There were no significant differences between physical performance variables depending on the match outcome. However, xGA (0.70 ± 0.39 vs. 1.24 ± 0.59; *p* = 0.004) and xGCA (5.38 ± 2.78 vs. 10.92 ± 6.18; *p* = 0.002) were significantly lower in wins than in matches that ended in losses (Table 2).

**Table 2:** Physical (m) and technical-tactical performance according to the outcome (mean ± SD).

		Match outcome		
		Win	Draw	Loss
Physical Performance Measures	TD (m)	10931 ± 371.7	10809 ± 481.3	10862 ± 662.7
	TD 20–25 (m)	658 ± 55.8	648 ± 76.9	664 ± 77.5
	TD >25 (m)	226 ± 28.9	209 ± 43.6	236 ± 56.3
	TD >20 (m)	884 ± 74.8	857 ± 108.8	899 ± 132.2
	D-ACC (m)	222 ± 17.2	214 ± 28.2	229 ± 20.2
	D-DEC (m)	218 ± 14.2	211 ± 26.6	217 ± 22.4
Technical-Tactical Performance Measure	xG	1.698 ± 0.944	1.404 ± 0.971	1.251 ± 0.806
	xGA	0.704 ± 0.390^$^	0.806 ± 0.622	1.239 ± 0.591
	xGC	16.188 ± 12.665	11.788 ± 7.325	13.735 ± 7.201
	xGCA	5.384 ± 2.782^$^	7.580 ± 6.959	10.917 ± 6.183
	PPDA	6.814 ± 0.977	6.238 ± 1.122	6.571 ± 1.224

TD = Total Distance, TD 20–25 = Distance covered between 20 and 25 km/h, TD>25 = Distance covered above 25 km/h, TD>20 = Distance covered above 20 km/h, D-ACC = Acceleration Distance above 3 m/s/s, D-DEC = Deceleration Distance above 3 m/s/s. xG = Expected Goal, xGA = Expected Goal Against, xGC = Expected Goal Chain, xGCA = Expected Goal Chain Against, PPDA = Passes Per Defensive Actions. ^$^ (*p* ≤ 0.05): significant difference between a win and a loss (*p* ≤ 0.05) after one-way ANOVA and post hoc analysis (Bonferroni test).

The correlation analyses between the physical performance and the technical-tactical performance are reported in Table 4. There were weak significant correlations between xGCA and D-ACC and between xGCA and D-DEC (Table 3).

**Table 3:** Correlation matrix between technical-tactical performance and physical performance.

			Technical-Tactical Performance Measures				
			xG	xGA	xGC	xGCA	PPDA
**Physical Performance Measures**	**TD**	Pearson’s r	−0.089	0.057	−0.014	−0.000	0.155
		*p*-value	0.462	0.635	0.909	0.999	0.196
		Upper bound of the 95% CI	0.148	0.287	0.220	0.233	0.375
		Lower bound of the 95% CI	0.315	−0.178	−0.246	−0.233	−0.081
	**TD 20–25**	Pearson’s r	−0.036	0.171	−0.055	0.104	−0.032
		*p*-value	0.765	0.154	0.650	0.387	0.794
		Upper bound of the 95% CI	0.199	0.389	0.181	0.330	0.203
		Lower bound of the 95% CI	−0.267	−0.065	−0.284	−0.132	−0.263
	**TD >25**	Pearson’s r	0.118	0.204	−0.004	0.219	0.035
		*p*-value	0.329	0.089	0.974	0.067	0.773
		Upper bound of the 95% CI	0.342	0.417	0.230	0.430	0.266
		Lower bound of the 95% CI	−0.119	−0.031	−0.237	−0.015	−0.200
	**TD >20**	Pearson’s r	0.024	0.199	−0.039	0.160	−0.007
		*p*-value	0.842	0.097	0.749	0.181	0.957
		Upper bound of the 95% CI	0.256	0.413	0.196	0.380	0.227
		Lower bound of the 95% CI	−0.210	−0.036	−0.270	−0.076	−0.240
	**D-ACC**	Pearson’s r	−0.092	0.160	−0.109	0.255*	0.054
		*p*-value	0.446	0.184	0.363	0.032	0.655
		Upper bound of the 95% CI	0.145	0.379	0.127	0.461	0.284
		Lower bound of the 95% CI	−0.318	−0.077	−0.334	0.023	−0.182
	**D-DEC**	Pearson’s r	−0.104	0.192	−0.155	0.237*	0.062
		*p*-value	0.387	0.109	0.197	0.047	0.607
		Upper bound of the 95% CI	0.132	0.407	0.081	0.445	0.291
		Lower bound of the 95% CI	−0.329	−0.043	−0.375	0.003	−0.174

TD = Total Distance, TD 20–25 = Distance covered between 20 and 25 km/h, TD>25 = Distance covered above 25 km/h, TD>20 = Distance covered above 20 km/h, D-ACC = Acceleration Distance above 3 m/s/s, D-DEC = Deceleration Distance above 3 m/s/s. xG = Expected Goal, xGA = Expected Goal Against, xGC = Expected Goal Chain, xGCA = Expected Goal Chain Against, PPDA = Passes Per Defensive Actions. * *p* ≤ 0.05

## Discussion

This study aimed to compare physical and novel technical-tactical performance indicators between matches with different outcomes for a soccer team competing in the French Ligue 2 championship during the 2020/2021 and 2021/2022 seasons. The results of this study are consistent with the initial hypotheses. It was found that there were no significant differences in the physical performance variables based on the match outcome. However, significant differences in technical-tactical performance variables between matches with different outcomes were found (xGA and xGCA were significantly lower in wins compared to matches ending in losses). Overall, the data of this study suggest that the reduction in the opposing team's goal expectancy may be associated with a better match outcome (i.e., victory), although the limited sample size and statistical methods used indicate that causality cannot be inferred.

Our findings suggest that technical-tactical factors associated with goal expectancy have an effective impact on the match outcome, especially when the team was effective in reducing the number and quality of the shooting opportunities of the opposing team. More specifically, low xGA and xGCA scores denote a more efficient defense against rival shooting, which significantly increases the probability of winning the match; in contrast, higher physical running performance metrics do not seem to have an impact on the match outcome. Additionally, a high xGCA score was positively associated with the distance in acceleration and deceleration, indicating that the team under investigation was required to accelerate/decelerate greater distances when it faced an opposing team with higher values of expected goals. This suggests that the increased acceleration/deceleration distance was a response to the opposing team with higher attacking power. Collectively, all this information indicates that coaches should implement technical-tactical strategies to reduce the shooting capacity of the rival as low values of xGA and xGCA will increase the likelihood of winning matches. Additionally, professional soccer teams should be prepared to perform more accelerative and decelerative distances when facing rivals with higher attacking abilities.

Previous studies in elite soccer showed no consensus for the association between physical performance and the match outcome. Different authors have shown higher running performance in wins (Chmura et al., 2018, 2022; Aquino et al., 2017, 2020; Gonçalves et al., 2021; Schauberger et al., 2018). In contrast, other research has shown higher run values during losses (Augusto et al., 2022). Moreover, both successful and unsuccessful teams may have very similar running performance as shown in other studies (Asian-Clemente et al., 2019; Aquino et al., 2021). The results of this study confirm data from previous investigations in which players had similar running metrics irrespective of the final match outcome. This information suggests that running at high and very high speed can be as important in wins as in losses as both decisive attacking and defensive actions during the match are performed at high intensity. This should not be interpreted as if the running metrics do not impact the match outcome, but as confirmation that high running performance is key for soccer games, irrespective of the match outcome. In this context, future investigations on running performance during soccer should include vectors to assess running directions to assess when a player is running towards the rival goal (as it likely will denote an offensive action) vs. when the player is running towards his own goal (as it likely will denote a defensive action). This characterization will help understand what type of running actions are more common when winning/drawing/losing matches.

Some technical-tactical aspects examined in this study show stronger relationships with the match outcome, especially those associated with the goal expectancy of the opposing team. Previous investigations have suggested that there are several technical indicators with a better predicting power of team's success (Clemente et al., 2016). Among them, the number of shots, the number of shots at the target, the number of shots from open play, shooting from counterattack and the percentage of ball possession have been defined as key performance indicators (KPI) (Lago Ballesteros and Peñas, 2010; Lago-Peñas et al., 2010; Peñas and Lago Ballesteros, 2011). Interestingly, the results of this study are novel because our data indicate that some technical-tactical variables associated with the defensive capacity of the team (xGA and xGCA) are also linked to the probability of victory. Additionally, due to the nature of the current analysis, we can also show that these technical-tactical factors are better than physical factors in predicting the outcome of a match for a professional soccer team. xGA and xGCA scores were higher in matches that ended in losses, which implies that when a team shows high values of xGA and xGCA, the probability of losing the match increases. Although these variables are designed to assess the capacity of the opposing team to generate shooting actions, they also indirectly refer to the capacity of the own team to defend. Thus, high xGA and xGCA scores during a match may be the product of a highly effective attacking team in terms of shooting, a poorly effective defense team in terms of blocking shooting actions or a combination of both. A possible reason for these results may be that these technical-tactical factors are strongly correlated to the number of goals conceded (Anzer and Bauer, 2021). Additionally, we found that a high xGCA score was positively associated with the distance in acceleration and deceleration. We also found that a high xGCA score was positively associated with acceleration and deceleration distance. Thus, we can deduce that physical actions such as accelerations and decelerations are more useful during defensive phases (as pressure on an opponent).

Technical-tactical aspects of soccer are more important than physical aspects to have a good match outcome (win). The priority for the coach will therefore be to know how to optimize the technical and tactical aspects. Firstly, it is necessary for the coach to have the maximum number of available players to train the technical and tactical aspects with the most complete squad possible. Another consideration is the reduction of the rate of injury which is an important factor for the team success in the championship (Eirale et al., 2013) and obviously for the staff to have a maximum number of players to work with during the training sessions. Secondly, we note that technical and tactical aspects must be a priority for the staff in the planning and construction of their training content. Indeed, as the technical and tactical aspects are more decisive compared to physical factors for achieving victories, practitioners need to prioritize these activities during training.

Additionally, all wins were categorized within the same group irrespective of the goals scored or the difference of goals with respect to the opposing team. Investigations with more teams may allow the subcategorization of wins depending on the number of goals scored (or goal difference) to understand if performance indicators also vary depending on these variables. Secondly, we selected a high-ranked team from Ligue 2 and therefore, a low number of defeats (n = 13, 18.3% of the total matches played) occurred during the matches analyzed. This is a strength of the study as it includes the analysis of a successful team and determines which performance indicators may be key for soccer performance. Further investigations should analyze the impact of running metrics and technical-tactical variables on the match outcome in teams with a comparable number of victories/defeats.

The comparison of physical and technical-tactical indicators in wins/draws/losses should be performed in teams with different levels and countries as the association of performance indicators with victory may be affected by the team’s ranking.

In different countries, varying styles of play may predominate, resulting in differences in performance indicators and strategies. For example, in England, a fast-paced attacking game is often favored, emphasizing speed, directness, and physicality. On the other hand, in Italy, a more passing-based game with an emphasis on tactical discipline and control may be prevalent.

When analyzing performance indicators in different countries or leagues, it is essential to consider these variations in playing styles. Comparative studies can provide valuable insights into the unique characteristics and strategies employed by teams in different soccer cultures. By examining performance indicators within these contexts, researchers can better understand the factors that contribute to success and tailor their analyses accordingly.

Therefore, future investigations should consider the specific playing styles and tactical approaches of different countries or leagues to gain a comprehensive understanding of performance indicators and their implications. This comparative analysis can enrich our knowledge of the sport and contribute to the development of nuanced strategies and training methodologies specific to each soccer culture.

One final point, the data of players who did not play the full 90 minutes in matches were not considered. Future research could include the data of these players by utilizing physical performance ratios in meters per minute (m/min) to encompass all players participating in the matches.

## Limitations

The current study has some limitations: i) the team was professional, but played on a national second league. The study of these variables in higher-performance teams competing in first-division of national leagues or international competitions merits further investigation. ii) We only analyzed a few of the hundreds of new technical-tactical variables that have appeared in the last years. Further investigations should be conducted to include more of these modern variables, especially the ones that include calculations of the interactions between players. iii) This study was carried out with male players and its results should not be applied to women’s soccer.

## Conclusions

Our study shows that better results of some technical-tactical variables were obtained when the team obtained a victory, especially those that reduced goal expectancy of the rival team. On the contrary, running performance metrics were not associated with the match outcome, likely because high running performance values are required in both wins and losses. From a practical perspective, soccer coaches should implement technical-tactical strategies to increase the teams’ expected goal statistics while avoiding opponent’s strategies to obtain clear shooting opportunities.
